# Early diagnosis and care is achieved but should be improved in infants with Prader-Willi syndrome

**DOI:** 10.1186/s13023-017-0673-6

**Published:** 2017-06-28

**Authors:** Céline Bar, Gwenaelle Diene, Catherine Molinas, Eric Bieth, Charlotte Casper, Maithé Tauber

**Affiliations:** 1 Endocrinology, Obesity, Bone Diseases, Genetics and Gynecology Unit, Children’s Hospital, University Hospital Center of Toulouse, 330, avenue de Grande-Bretagne, TSA 40031 – 31059 Toulouse cedex 9, France; 2 National Reference Center of Prader-Willi Syndrome, Children’s Hospital, University Hospital Center of Toulouse, 330, avenue de Grande-Bretagne, TSA 40031 – 31059 Toulouse cedex 9, France; 3 Genetics Unit, University Hospital Center of Toulouse, Toulouse, France; 4 Neonatology Unit, Children’s Hospital, University Hospital Center of Toulouse, 330, avenue de Grande-Bretagne, TSA 40031 – 31059 Toulouse cedex 9, France; 5 Centre de Physiopathologie de Toulouse-Purpan, Université de Toulouse, CNRS UMR 5282, INSERM UMR 1043, Paul Sabatier University, Toulouse, France; 6 Genetics Unit, Institut Fédératif de Biologie (IFB), 330, avenue de Grande-Bretagne, TSA 40031 – 31059 Toulouse cedex 9, France

**Keywords:** Prader-Willi syndrome, Neonatal care, Birth incidence, Early diagnosis, Prenatal diagnosis, Delayed diagnosis

## Abstract

**Background:**

PWS is a severe neurodevelopmental genetic disorder now usually diagnosed in the neonatal period from hypotonia and feeding difficulties. Our study analyzed the birth incidence and care of infants with early diagnosis.

**Methods:**

Data were collected on 61 infants with a molecular diagnosis of PWS born in 2012 and 2013 in France.

**Results:**

Thirty-eight infants with PWS were born in 2013. The median age at diagnosis was 18 days. Birth incidence calculated for 2013 was 1/21,000 births. No case was diagnosed prenatally, despite 9 amniocenteses, including 4 for polyhydramnios. Five infants had delayed diagnosis, after 3 months of life. For 2 of them, the diagnosis was not suspected at birth and for 3, FISH analysis in the neonatal period was normal, with no further molecular studies. Ninety-three percent of the neonates were hospitalized, and 84% needed nasogastric tube feeding for a median of 38 days. Swallowing assessment was performed for 45%, at a median age of 10 days. Physiotherapy was started for 76% during hospitalization. Eighty percent of those diagnosed within the first 3 months were seen by a pediatric endocrinologist within the first week of life.

**Conclusion:**

Our study is the first to assess the birth incidence of PWS in France, at 1/21,000 births. Some prenatal or neonatal cases remain undiagnosed because of unrecognized clinical signs and the inappropriate choice of the initial molecular test. We also underscore the need to optimize neonatal care of infants with PWS.

## Background

Prader-Willi syndrome (PWS) was first described in 1956 by Prader, Labhart and Willi [1]. It is a complex neurodevelopmental genetic disorder which associates severe hypotonia and feeding difficulties with sucking deficit and anorexia in the neonatal period [2]. In the absence of early diagnosis and care, excessive weight gain occurs and several nutritional phases have been described, leading to severe early obesity starting between 3 and 4 years of age [3, 4].

Diagnosis is possible within the first months of life because these infants display severe hypotonia, and neonatologists recognize this sign alone as sufficient to suggest the need for genetic study [5–7]. Early diagnosis and adequate multidisciplinary care are crucial for the infant’s outcome as they ensure parental guidance and support, including comprehensive advice to prevent obesity, and stimulation of cognitive and adaptive skills [5]. In addition, we very recently reported for the first time the positive effects of an early short course of oxytocin treatment in infants with PWS younger than 5 months [8]. It is likely that the window of opportunity to obtain significant change is quite narrow, particularly regarding early brain plasticity, and delayed diagnosis may mean a missed opportunity for better quality of life in infants with PWS [8].

The genetic defect is a lack of paternal copy expression of some of the genes at the q11–13 locus of chromosome 15. Several genetic subtypes have been reported: deletion (DEL) in about 65% of the cases, maternal uniparental disomy (UPD) in about 30%, imprinting center defect in less than 5%, and rare cases of translocation involving the chromosome 15 q11-q13 region [9, 10]. Genetic diagnosis is now easily available for neonatologists who care for neonates with severe hypotonia.

The aims of this study were to determine the neonatal incidence of PWS in France and address the issues of delayed diagnosis. We also investigated the care of those newborns and infants who were diagnosed soon after birth.

## Methods

This study was conducted by the French PWS Reference Center (PWSRC), which is coordinated by the Toulouse University Hospital in association with the Neonatology Unit of the Children’s Hospital of Toulouse. We included those infants born in France between 1 January 2012 and 31 December 2013 with a molecular diagnosis of PWS. After several attempts to obtain comprehensive data, all contacts were asked to focus their attention on 2013 to ensure a maximal dataset for this year. The incidence was therefore only calculated for 2013, although the data on infant care were analyzed for 2012 and 2013. The birth incidence was obtained by dividing the number of PWS diagnoses by the number of live births in 2013, according to the French INSEE (National Statistics Institute) database.

Neonatology units and PWS competence centers were contacted through the French Neonatology Society and the French Pediatric Endocrinology and Diabetology Society. Data obtained by the French Prader-Willi Association (FPWA) were also collected. Based on information from the Orphanet website, we also contacted by email all cytogenetic and molecular biology laboratories that perform PWS diagnosis in France to ensure that every prenatal and neonatal infant born during this period was counted. The first three letters of the first and last names and the date of birth identified cases and avoided duplicates.

Neonatologists and pediatricians were asked to report data about their own PWS patients on a clinical report form (CRF) sent by email. The requested information concerned the pregnancy, neonatal period, diagnosis, initial care and family data. Neonates with birth weight and/or height below −2 standard deviation scores (SDS) according to Usher and MacLean’s tables [11] were considered as small for gestational age (SGA). The results are expressed as medians, 10th and 90th percentiles (10pc; 90pc) and/or mean values ± SDS. Statistical analyses were made with Statview software. The Chi2 test compared proportions and the Mann-Whitney U-test compared the quantitative data.

## Results

### Incidence of PWS at birth in 2013

In metropolitan France, 781,621 live births were reported in 2013, of which 38 infants had a molecular diagnosis of PWS. The 2013 birth incidence was therefore 1/20,778. We also identified 3 prenatal diagnoses in 2013, 2 of which led to pregnancy interruption. The outcome of the third pregnancy was unknown.

### Age at diagnosis in neonates

**Fig. 1:**
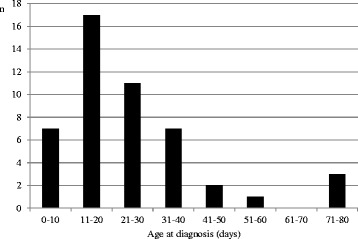
Age at molecular diagnosis of infants with PWS diagnosed before 3 months of life (*n* = 48)

In 2013, the median age at diagnosis was 18 days (5th percentile, 7; 95th percentile, 50) including the 2 late diagnoses. For the 23 infants retrieved in 2012 for our survey, the median age was 23 days. These 61 infants showed a wide range of values (Fig. 1). The median delay between blood sampling and genetic results was 10 days. As shown in Fig. 1, 90% of the infants were diagnosed before the third month of life.

### Genotype subtypes

The genotype subtype was known for 57/61 patients (93%). Four infants had an abnormal methylation profile confirming the diagnosis of PWS, with no further molecular study at the time of the survey. Fifty-three percent of the neonates had a DEL, 42% had a UPD, 3% had a translocation, and 2% had an imprinting center defect.

Mean maternal age at birth of the PWS infant was 33 years, and 32 years when it was the first child. Mean paternal age at birth of the PWS infant was 36 years, and 34 years when it was the first child. Maternal and paternal ages were significantly higher in neonates with UPD compared with neonates with no UPD (38 years versus 31 years, *p* = 0.0001 and 38 years versus 33 years, *p* = 0.003, respectively).

### Pregnancy, amniocentesis and birth data

The CRFs were filled out by pediatric endocrinologists (42%), neonatologists (17%), geneticists (12%), pediatricians (12%) and residents (11%). The findings of all 61 infants with PWS are detailed in Table 1.

**Table 1:** Prenatal and neonatal characteristics of newborns with PWS (*n* = 61)

	N = Median (10pc; 90pc)	Mean (±SDS)
Sex (Male/Female)	28 (46%)/33 (54%)	
Gestational age (WA)	39 (35;42)	39 ± 3.1
MAP	3/54 (5.5%)	
Decreased fetal movements	15/56 (27%)	
Polyhydramnios	12/53 (23%)	
Amniocentesis	9/46 (20%)	
Preterm birth <37 WA	12/59 (20%)	
Cesarean section	39/58 (67%)	
APGAR score 1 min	9 (4;10)	7.6 ± 2.7
APGAR score 5 min	10 (7.5;10)	9.2 ± 2
Birth weight (SDS)	-1.2 (-2.2;-0.2)	-1.2 ± 0.8
Birth length (SDS)	-1.2 (-2.3;-0.1)	-1.1 ± 1
Birth head circumference (SDS)	-0.2 (-2.2;1.1)	-0.5 ± 1.4
Small for gestational age	17/56 (30%)	

SDS: standard deviation score. WA: weeks of amenorrhea. MAP: Medically assisted procreation

Twelve neonates (20%) were born preterm, 6 with DEL, 5 with UPD and one with translocation. Three infants (2 with DEL and 1 with UPD) were born after medically assisted procreation (MAP): one in-vitro fertilization, one ovarian stimulation and one intracytoplasmic sperm injection.

Nine amniocenteses were performed and the indications and results are shown in Table 2. There was no pregnancy interruption based on the results of amniocentesis.

**Table 2:** Indications and results of the nine amniocenteses

Case	Sex	Indication	Result	Postnatal genotype
2	F	Maternal age, clubfoot, nasal bone abnormality	Normal karyotype	Abnormal methylation profile
6	F	Hydramnios, IUGR	Normal karyotype, no PW deletion	UPD
15	F	Hydramnios, IUGR, hypomobility	Normal karyotype	Deletion
43	F	Hydramnios	Normal karyotype	UPD
45	F	Cervical hygroma	Normal karyotype	Deletion
46	M	IUGR, VSD, hypospadias	Normal karyotype, negative 22q11 FISH	UPD
47	M	Abnormal combined test	Normal karyotype	Deletion
50	F	Abnormal combined test	Normal karyotype	UPD
52	F	Hydramnios	Normal karyotype	Deletion

IUGR: intrauterine growth retardation, VSD: ventricular septal defect, UPD: uniparental disomy. The combined test for systematic screening for Down syndrome is performed between 10 weeks and 13 weeks and 6 days of amenorrhea, according to maternal age; it comprises ultrasound measurement of nuchal translucency and plasma evaluation of free β-human chorionic gonadotropin and pregnancy-associated plasma protein A (PAPP-A)

### Delayed diagnoses

Five patients were diagnosed after 3 months of life. Two were diagnosed at 11 and 17 months, respectively, based on cryptorchidism with hypoplastic penis noted by a surgeon and psychomotor delay. The diagnosis had not been evoked in neonatology for these 2 infants. In the other 3 infants (43, 48 and 61), diagnosis was evoked in the neonatal period but the fluorescent in situ hybridization (FISH) tests were normal. Patient 43 had a mosaic UPD. The initial FISH test results were therefore normal. Further exploration with methylation analysis, comparative genomic hybridization (CGH), and single nucleotide polymorphism (SNP) array was then carried out, causing the delay in diagnosis, which was made at 6.5 months. For patients 48 and 61, diagnosis was made at 5 and 24 months, respectively, based on clinical features suggesting PWS.

Data on karyotypes were obtained for 49/61 infants. Karyotyping was performed for 42 infants and was normal for 33 (79%). Among the 9 abnormal karyotypes, 7 had DEL, one had an unbalanced 9;15 translocation, and one had a Robertsonian translocation that was subsequently diagnosed as UPD.

### Announcement of the diagnosis

Data about announcing the diagnosis to parents were obtained for 47/61 infants. The diagnosis was announced by two physicians in 45% of the cases and in the presence of a geneticist and/or a neonatologist in 60% of the cases. The pediatric endocrinologist was present in 17% of the cases.

### Neonatal care

The data indicated that 57/61 neonates (93%) had been hospitalized, 43 of them (75%) right after birth and 11 later on with a median delay of 5 days. Median hospital stay was 32 days. Data were missing for 3 patients. In this section, the number of infants analyzed is specified for each criterion because of differences in missing data.

*Feeding problems:* Data about feeding were obtained for 55 newborns: 26 were completely or partially breastfed or fed with milk from the hospital milk bank for a median of 30 days (7.2; 156). Data about nasogastric tube (NGT) feeding were obtained for 58 newborns: 49 required NGT feeding for a median of 38 days (10; 150). No patient had a gastrostomy. Data on swallowing assessment by a swallowing specialist were obtained for 40 infants: 18 were assessed at a median age of 10 days (5.4; 30).

*Hypotonia:* We obtained data about physiotherapy during hospitalization for 42 infants: 32 received physiotherapy.

*Respiratory problems:* We obtained data about intubation for 49 newborns: 6 were intubated, and 67% of these infants were preterm. Median duration of intubation was 16 days (5.5; 34.5). Data about noninvasive ventilation were obtained for 42 newborns: 14 (33%) were treated with continuous positive airway pressure (CPAP), and 43% of these infants were preterm. The median duration of CPAP was 13.5 days (2.3; 96.6). Data about nasal oxygenotherapy were obtained for 49 newborns: 16 required it and 31% were preterm. The median duration was 5 days (1; 19.6).

There was no difference in care between the different genetic subtypes.

### Hospital discharge and rehabilitation

Seventy-eight percent of the newborns were discharged straight to their homes and 18% benefited from neonatology home hospitalization (HH) for a median of 35 days. Home hospitalization enables the safe continuation of adapted newborn care in the home so that families can return home more quickly. Four percent were transferred to another hospital unit. Newborns with HH had been diagnosed earlier than those without it (17 days versus 27 days, *p* = 0.04). However, the mean hospital stay did not differ between the two groups: 39 days for patients with HH (*n* = 8) and 30 days for those without it (*n* = 32, *p* = 0.37).

On discharge, 44 infants had prescriptions for physiotherapy, 21 for psychomotor care, and 21 for speech therapy, out of, respectively, 51, 37 and 47 infants for whom we had data.

### Pediatric endocrinology follow-up

**Fig. 2:**
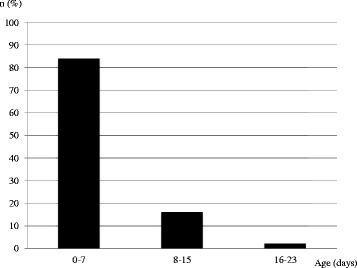
Age at first visit with a pediatric endocrinologist of infants diagnosed with PWS before 3 months of life (*n* = 43)

The first visit with a pediatric endocrinologist was made at a median age of 3 months (1; 12). The distribution of age at this first visit is shown in Fig. 2 for the 43 patients diagnosed within the first 3 months of life. In this subgroup, more than 80% were seen within the first week of life: 67% in a regional hospital and 24% in the PWSRC.

For the whole group of 61 infants, 53 (87%) had at least one visit with a pediatric endocrinologist during the first 2 years, with 80% before 6 months of life.

*Start of growth hormone (GH) treatment:* Median age at study inclusion was 18.8 months (9.3; 27.9). Data for GH treatment were obtained for 51 infants and 22 of them had started GH. Mean age at the start of GH treatment was 12 ± 4.5 months (7–23). Those who did not start were younger (17 months versus 24 months, *p* = 0.007).

### Knowledge about the Reference Center and the patients’ association

Thirteen out of the 38 clinicians (34%) who filled in a CRF were aware of the Reference Center document about the announcement of diagnosis to parents, available on the Reference Center website. Forty-three of 45 neonatologists (96%) knew about the PWSRC, 28/40 (70%) knew about the PWSRC website, 41/44 (93%) knew about the FPWA, and 16/39 (41%) knew about FPWA website.

## Discussion

*Birth incidence and genetic diagnosis:* We show for the first time that the birth incidence of PWS in France in 2013 was about 1/21,000 births. This is higher than the incidences reported in early 2000 in Australia and Belgium [12, 13], which were about 1/27,000 births, but lower than that reported by Lionti in 2014, which was 1/15,830 births between 2003 and 2012 [14].

In our study, the median age at diagnosis was 18 days in 2013. To our knowledge, this is the earliest age reported so far: one month for Smith [12] and 6.5 months for Vogels [13]. Given that the mean delay between blood sampling and results was 10 days, our data indicate that the diagnosis was suspected within the first week of life. The range of values was quite wide, with most patients (90%) diagnosed within the first 3 months and 5 diagnosed later. Diagnosis was delayed for 2 main reasons: either it was not suspected in neonatology or FISH as the first-line genetic test gave false negative results. This last underlines the risk of performing FISH first.

Although our study shows that the mean age at diagnosis was 18 days, which is very good, we also found delayed diagnoses, suggesting that systematic neonatal screening for PWS should be considered to prevent neonates with PWS from being missed. Indeed, the criteria required to set up neonatal screening for PWS have been met: we report a neonatal incidence close to that of congenital adrenal hyperplasia (1/19,000), for which screening has been performed for many years; the diagnostic tools are well known and reliable (methylation profile abnormality positive in 99.99% of cases of PWS); and early diagnosis enables multidisciplinary care to begin quickly, improving prognosis and providing a window of opportunity for early treatment that may alleviate and change the course of the disease [5]. In addition, Bachere et al. [5] noted that early diagnosis and multidisciplinary care reduces the hospital stay and the duration of tube feeding, optimizes screening for endocrine dysfunction, and prevents growth delay and the early onset of obesity.

We confirmed a previous report [15] of a rise in the proportion of UPD, up to 42% compared with older studies. Recently, Beauloye et al. [16] reported a UPD proportion of 51% in children from 1 to 48 months old and, notably, this genetic subtype was associated with advanced maternal age [17]. We found similar data in our study, with maternal age of UPD being 38 years versus 31 years for DEL (*p* = 0.0001). Moreover, the mean maternal age of the whole cohort was higher than in the general population (32 years for a first child versus 28 years in the general population, according to INSEE data). As already reported [18, 19], the median paternal age was also older for UPD than for DEL cases (38 versus 33 years, *p* = 0.003).

In our study, the MAP rate was 5.5%, which is twice that reported in the general population (2.9% in 2012 according to the Biomedicine Agency [20]). However, no patient in our study had epigenetic mutation, which is more frequent in MAP-induced imprinting diseases [21].

*Challenges for prenatal diagnosis:* Incidences of decreased fetal movement (27% in our study) and polyhydramnios (23%) were less frequent than previously reported (88% and 34%, respectively, for Gross et al. [22]; 91% and 36%, respectively, for Geysenbergh et al. [23]). However, even in cases of these prenatal signs, the amniocenteses reported in our study did not lead to the prenatal diagnosis of PWS. Indeed, out of 9 amniocenteses, 4 were performed for polyhydramnios (alone or associated with other signs), while genetic screening for PWS was performed in only one case with FISH, which gave a normal result. No further molecular analysis was made to exclude or confirm the diagnosis. These data suggest that prenatal diagnosis might be optimized by considering PWS testing with methylation profile analysis when polyhydramnios is present.

*Challenges for neonatal care:* Most neonates needed NGT feeding (84%) for a median of 38 days, as already reported [5]. Only 18/40 infants had a swallowing assessment, and this evaluation should be promoted. It is performed and interpreted by a speech and language expert, who is a critical caregiver for infants with PWS. At the end of hospitalization, only 21/47 infants had a prescription for speech and language therapy, whereas 44/51 infants had prescriptions for physiotherapy and 21/37 for psychomotor therapy. This study underscores the need to improve the evaluation of oral skills and prescription of speech and language therapy for these newborns, in addition to physiotherapy or psychomotor therapy.

*Endocrine follow-up:* The first visit to a pediatric endocrinologist was made within the first 7 days of life for 84% of the infants diagnosed within the first 3 months. For the whole cohort, the median age at the first visit was 3 months. Endocrine dysfunctions could thus be detected early and GH treatment started within the first year, as recommended [24]. Indeed, 43% of the infants had already started GH treatment at the time of inclusion, with a mean age of 12 months, which is excellent, given the GH effects on muscle and cognitive development in these patients [25].

## Conclusion

Our study is the first to assess the birth incidence of PWS in France, at about 1/21,000 births. We confirm the increasing proportion of UPD in conjunction with advanced maternal age. Prenatal and neonatal cases are still sometimes missed, generally because the early signs are not recognized or inappropriate molecular tests are prescribed and may therefore give false negative results. Setting up neonatal PWS screening procedures would prevent these late diagnoses and permit early care and treatment. Comprehensive neonatal care should include a systematic sucking and swallowing assessment in order to optimize feeding and mother-infant interaction.

## Abbreviations

CGH: Comparative genomic hybridization
CNIL: French National Commission for Information Technology and Civil Liberties
CPAP: Continuous positive airway pressure
CRF: Clinical report form
DEL: Deletion
FISH: Fluorescent in situ hybridization
FPWA: French Prader-Willi Association
GH: Growth hormone
HH: Home hospitalization
INSEE: National Statistics Institute
IUGR: Intrauterine growth retardation
MAP: Medically assisted procreation
NGT: Nasogastric tube
PAPP-A: Pregnancy-associated plasma protein A
PWS: Prader-Willi syndrome
PWSRC: Prader-Willi Syndrome Reference Center
SDS: Standard deviation score
SGA: Small for gestational age
SNP: Single nucleotide polymorphisms
UPD: Uniparental disomy
VSD: Ventricular septal defect
WA: Weeks of amenorrhea
